# To make a difference – how GPs conceive consultation outcomes. A phenomenographic study

**DOI:** 10.1186/1471-2296-10-4

**Published:** 2009-01-16

**Authors:** Annika Andén, Sven-Olof Andersson, Carl-Edvard Rudebeck

**Affiliations:** 1 Bergnäsets Vårdcentral, Luleå, Sweden; 2 Department of Medical and Health Sciences, Inst for Community medicine/General practice, Linköping University, Linköping, Sweden; 3 Department of Public Health and Clinical Medicine, Umeå University, Umeå, Sweden; 4 Kalmar County Council, Vårdcentralen Esplananden, Västervik, Sweden; 5 Department of Community Medicine, Tromsö University, Tromsö, Norway

## Abstract

**Background:**

Outcomes from GPs' consultations have been measured mainly with disease specific measures and with patient questionnaires about health, satisfaction, enablement and quality. The aim of this study was to explore GPs' conceptions of consultation outcomes.

**Methods:**

Interviews with 17 GPs in groups and individually about consultation outcomes from recently performed consultations were analysed with a phenomenographic research approach.

**Results:**

The GPs conceived outcomes in four ways: patient outcomes, GPs' self-evaluation, relationship building and change of surgery routines.

**Conclusion:**

Patient outcomes, as conceived by the GPs, were generally congruent with those that had been taken up in outcome studies. Relationship building and change of surgery routines were outcomes in preparation for consultations to come. GPs made self-assessments related to internalized norms, grounded on a perceived collegial professional consensus. Considerations of such different aspects of outcomes can inspire professional development.

## Background

GPs' consultation outcomes have, to a great extent, been evaluated by measurable illness parameters such as HbA1c or blood pressure. As GPs meet all sorts of patients with all sorts of problems there has also been a need to understand and systematize GPs' consultation outcomes without relating them to diagnoses, disease or illness. For this, non-disease-specific outcome measures have been used. Most used are satisfaction instruments such as MISS [1] and CSQ [1] and health instruments such as SF-36 [2] or EQ5D [3,4]. An instrument for measuring if the patient has been enabled to cope with illness and life is Patient Enablement Instrument, PEI [5].

During the last decades quality assessment has been increasing. The Europep studies [6] as well as the instruments GPAQ [7] and IPQ [8] that are currently used in UK, are examples. They contain questions about the doctor's professional performance and attitudes, care settings and consultation outcomes.

Thus, there is great variation both as to parameters and to methods, when GPs' consultation outcomes are being studied. An important question is then whether the measures or instruments are sensitive enough to capture a sufficient scope of possible outcomes. The questions in the health instruments are difficult to connect to the specific consultation outcome. The questions in the satisfaction instruments concern satisfaction with the doctor's attitudes and performance, and those, as well as the quality instruments, deal mostly with the settings and the process of care. The few outcome questions in the quality instruments are not discernible enough, even though the outcome might be expected to be the main item in evaluations. The consultation outcome could just as well be excellent as catastrophic, independent of whether the GP had been nice or the entrance had been handicap adjusted. Those limitations in our opinion make it important to develop more knowledge about GPs' consultation outcomes. It is likely that there may be consultation outcomes that have been overlooked in the instruments used today. In an earlier qualitative study of patients' experiences of consultation outcomes, additional aspects of outcome were found [9]. Now is the turn of the GPs'.

The GPs' experiences of consultation outcomes are not simply a complement to other perspectives but will also determine the actual outcomes. The GP will, in the consultation and in interaction with the patient, form a more or less clear goal for the consultation. This goal is a desirable and realistic outcome and it will be the objective of the consultation. Within the consultation, goal, course of action and outcome are linked together as practical knowledge [10]. Within this perspective, a concordance between measured outcomes and the ones that the GPs find significant will be crucial. In a British study from 2004 GPs described that lack of recognition for good work was a contributing factor to low job satisfaction [11]. A reasonable interpretation is that there are differences between GPs' perceptions of their achievements and what is being measured or registered.

Starting from the fact that the outcome measures and instruments being used have inevitable limitations and that most likely there are differences between the outcomes that GPs perceive and the ones being measured, the aim of this study was to explore GPs' conceptions of actual consultation outcomes.

## Methods

### Phenomenography

Phenomenography is a research approach developed in pedagogic research [12-15]. It originates from the observation that whatever phenomenon or situation people encounter they experience it in a limited number of distinctly different ways. The focus of the research is the different "ways of experiencing". This is called a second order perspective and it is different from a first order perspective, which describes things "as they are" [13]. What is experienced is most often described in statements, but it could also be for example, drawings or video-recordings. The statements (or other descriptive units) are sorted and grouped together in description categories. The description categories together form the outcome space, which is a picture of the phenomenon under investigation. Through examination, description and comparison of the different conceptions the phenomenon can be understood.

A phenomenon is discernible through its different aspects. Referential aspects refer to how the phenomenon relates to its surroundings, its global aspect. Structural aspects refer to the structure of the phenomenon, its characteristics. The structural aspect refers both to the way the parts of the phenomenon are delimited from and related to each other – the internal horizons and how the parts are delimited from their context – the external horizons [16].

As phenomenography uses different human experiences as a resource, it is a research approach suitable for health research, where a diversity of experiences can give a more thorough understanding of a phenomenon. General practice handles people with a diversity of experiences and it is important to be able to recognize them all.

Our object of research was GP's consultation outcomes seen through their eyes. The outcome of a consultation is different from the settings or the process of it. The settings are the circumstances surrounding the consultation, e.g. accessibility, and the process is what happens during the consultation, e.g. whether the doctor had been pleasant. The outcome is the change that has happened after the consultation and owing to it. This is the referential aspect of the consultation outcome.

### The GPs

Seventeen GPs from northern Sweden were interviewed, twelve in three groups and five individually. The selection was gradual and strategic to get a variation as to age, gender, ethnicity and years as a GP. The medium age of the GPs was 51(38–64). They had been working as GPs between six months and 28 years, nine were women and three had another mother tongue. They worked as public employees in group practices, which is the dominating type of employment for GPs in northern Sweden.

In order to gain the group's confidence already established groups, two CME groups and one group of experienced GPs from the same health centre, who had worked well together for a long time, were chosen. The group leader was contacted, who then asked the rest of the group to take part in the study.

### The interviews

The interviews were semi-structured. The group interviews were conducted by AA assisted by a male colleague and lasted one hour and a half. The individual interviews were conducted by AA and took about half an hour. Apart from a broader selection of GPs, the individual interviews gave us possibilities to see aspects that would eventually not come to light in group interviews.

The GPs were asked to describe the outcomes of their consultations. With the aim of getting unselected consultations, still fresh in their memory, they were asked to report on their most recent consultations. By associating the outcomes with actual patients, we wanted to find out how they perceived the factual outcomes of the actual patients, and not how they thought the outcomes ought to be.

As they had not earlier reflected over the consultation outcomes in a conscious way, the GPs found it interesting to do so. They expressed uncertainty and that it would partly be guesses. In the groups the other members helped the interviewers by asking the informants additional explanatory questions, but it was always the GP who had met the patient that formulated the conception of the outcome.

The GPs contributed with one to four cases each. The number was based on the length of the narratives and discussions. In the groups they were asked, one after the other. In all, 43 cases were reported, 25 from the groups and 18 from individual interviews. The cases were well spread as to age, gender, disease, illness and problems. This was noted as the GPs spontaneously reported their narratives as doctors usually describe cases, starting with age, gender, diagnoses and illness trajectory.

The interviews were tape-recorded and transcribed verbatim by AA. In addition careful notes were taken. The notes kept the main parts of the GPs' descriptions of the outcomes. A run-through with the sitter-in was performed immediately after the group interviews. The tape-recorder did not work in one group and one individual interview. Therefore the notes constituted the material for the analyses in those cases (in all thirteen cases, ten from a group and three from an individual interview). A preliminary analysis was performed after each interview. After the last it was assessed that little further information could be expected and the material was considered to be sufficient.

### The phenomenographic analysis

The analysis followed Sjöström's description of the seven steps of phenomenographic analysis [17]. The analysis started with repeated readings of the material. Statements considering outcomes from the actual consultations were picked out. One consultation could have several outcomes. The statements about outcomes were analysed, compared and sorted according to similarity into different groups. They were simply put into different heaps. The groups were given names as preliminary categories of description. These were compared with regard to similarities and differences both within and between the groups, and got their final names. The categories of description together form the outcome space. Finally a structure in the outcome space was identified.

## Result

The statements form four categories of description: patient outcomes, GPs' self-evaluation, relationship building and change of surgery routines.

Patient outcomes describe a goal for the consultation. GPs' self-evaluation is a reaction to the consultation. Relationship building is a basis for future consultations. A change of surgery routines is a change of the structure encompassing the consultation. These are all different ways to relate to the consultation outcome.

One consultation could have several outcomes but a statement was only referred to in one category.

The GPs started with a description of the patient outcomes and, after a little pause, they began to discuss other outcomes. Often this second part was opened with an evaluation of their own achievements.

### The goal for the consultation – patient outcomes

This category included what the GPs conceived had changed or would change for the patient due to the actual consultation. A future outcome was cure/symptom relief. Immediate outcomes were reassurance, increased understanding, support, check-up and satisfaction.

#### Cure/symptom relief

The GP expected the outcome to be cure or symptom relief through treatment or advice. In some cases a treatment or an action was necessary for cure. In others, a treatment would make the cure quicker and safer although recovery could be expected to be spontaneous.

Citation Dr M: *A woman in her fifties had a red swelling on her hand. I think she had erysipelas. I gave her penicillin. I think I will make her better faster this way*."

In our study the statements about cure were all about infections.

#### Reassurance

The GP experienced that the patient's worry about the symptom or condition had been reduced. The GP had reassured the patient by rejecting a specific disease or by explaining what caused the symptoms or by confirming that everything was being dealt with in the right way.

Citation Dr M:" *So I told him that nothing in the examination pointed at a haemorrhage or brain tumour. So I believe he left reassured*."

#### Increased understanding

The patient had received an explanation. The GP experienced that this lead to an increased understanding of what was happening in the patient's body or what to do to feel better. The patient had thus got increased knowledge.

Citation Dr Z:" *So I tried to give a reasonable explanation for her discomfort from the throat. You can have tensions there as well as in other muscles*."

Citation Dr T:"*He came with a locking in his thoracic. He had been seeing a chiropractor without results. He got better only when his new girl-friend gave him massage. So I asked him about the relationship and it turned out that the guy could not really decide if he wanted to continue or quit. This was an important outcome- that the ambivalence came up*."

#### A check-up

A check-up of a disease, or of risk factors, such as HbA1c or cholesterol gave information as to whether changes in therapy or follow-up were recommendable. The GP regarded it as his/her duty to see to it that the risk factors were under control. Sometimes he/she doubted that the patients could take their share of the responsibility. The tests and the disease were in focus even though the patient was well known to the GP. The GP said that he/she had done what should be done but did not express any further thoughts about possible consequences for the patient.

Citation Dr Y: *"Actually it is I who find it necessary to check up her diabetes, because she didn't have it under control."*

#### Support

The GP described that he/she gave support so the patient could cope with illness or life. The support could be the GP just being there without taking any action. It could also be a support to the patient to do something himself. In those cases it came close to increased understanding. It could also be substantial, as when sick-listing.

The patients who were perceived to have received support had long-lasting relations with their GPs. Mostly they had had psychiatric and/or musculoskeletal problems.

Citation Dr Y:" *I tell the patient that you are not alone. I will be beside you and if you need me you can just contact me."*

Citation Dr D:" *I help this unemployed man to stay on half-time sick-leave for a pain that is eternal, and thus I contribute to his daily living. Besides this, the only thing I can do for him is just to be there."*

#### Satisfaction

Sometimes the GP noticed that the patient seemed satisfied and sometimes the patient had expressed satisfaction. The comments on satisfaction were seen together with all other patient outcomes except check-up.

Citation Dr V:"*He seemed glad and pleased and shook my hand and thanked me."*

In its negative form there was only one statement saying that the visit was perceived as meaningless for the patient.

### A reaction to the consultation – GPs' self-evaluation

It became clear that it was important for the GPs not only to see patients' point of view, but also to perform good enough from the professional point of view. They had quite a determined interpretation of the professional perspective as to how to act as a GP in different situations. They presumed that these norms were shared by colleagues both inside and outside general practice. We found a picture of perceived professional norms regarding knowledge and values according to which they decided rightly and wrongly when evaluating their own achievements.

#### GP satisfaction

The GPs often stated that they were satisfied, which was expressed with words such as satisfied, nice, easy. Sometimes there followed a comment on what they were satisfied with, e.g. that they had succeeded or that they had inspired someone with confidence. They related their satisfaction to having done what they should; they had fulfilled their own expectations and were satisfied accordingly. When they believed in cure/symptom relief they often expressed that they were satisfied themselves.

Citation Dr M:" *It is nice to be a doctor when you feel you can make a difference."*

Citation Dr C:" *It was a good gut-feeling."*

#### Failure

When the GPs felt that they had not succeeded with the consultation they had an unpleasant feeling of failure. They had a concern that they had not lived up to the adopted professional norms. They blamed themselves. The colleagues in the group were however eager to give support in these situations.

The statements could deal with a lack of rapport with the patient, either when the GP felt persuaded to take an unnecessary action, or when the GP felt that the patient would not follow the recommendations given. These were situations where the GP simply could not do the right thing.

Citation Dr A:" *I have a bad feeling knowing that she doesn't look after her diabetes. Even though her glucose levels were high I didn't dare to increase her insulin as she doesn't manage her diet*. "

Citation Dr J:"*Big sister had got erythromycin for a mycoplasma infection and now her little sister had got the same sort of symptoms but milder. The mother was determined to get treatment for her. So I gave her antibiotics even though I didn't find it necessary, and I felt like shit. Dare I not stand up for anything?"*

Some accounts dealt with that the GP had performed an action that was necessary in the short perspective but maybe destructive in the long run.

Citation Dr T: "*an unemployed dyslectic man of 24, with headaches after whiplash traumas came for extended sick-listing, and got it. But it is a bad feeling to see him go towards a future with long sick-listing periods already from such young age."*

There were also examples of the GPs' worry about being despised by colleagues.

Citation Dr K: "*There was this four-year-old girl from a refugee family with haematuria. I sent her as an emergency to the paediatric clinic at four pm, mostly because of language difficulties. It was a really bad feeling. Of course it was best for the patient but not for the receiving clinic. During duty on the paediatric clinic they were always joking about the GPs and their stupid referrals."*

### A basis for future consultations – relationship building

The GPs described how they built up a relationship with the patient in different ways. They conceived the relationship building as an important outcome. In some cases the relationship was so important that their own ideas of the best treatment alternative were pushed into the background, especially when the patient had a very decided idea that was not particularly counterproductive. The relationship with the patient was of great importance for future consultations, but also in the current consultation.

Citation Dr O: "*He didn't get any medicines. We began by getting to know each other. We laid a basis for future consultations."*

Citation Dr V: "*She wanted physiotherapy for acne. I promised to make inquiries if there were any such treatments. You can try to meet her expectations even if it isn't what you would have suggested yourself if it isn't too bad of course. It can be worth it for future contacts with her."*

### A change of the structure embracing the consultation – change of surgery routines

In some cases the GP discovered a need to change surgery routines, which was also conceived as an outcome of the consultation.

Citation Dr R": *I discovered that it is not possible to have an HIV test anonymously in this place. We'll have to change that*."

## Discussion

### The Method

One strong point of the study was that there was a broad representation of both GPs and patients. They were representative for GPs and patients in Sweden, and probably more than that, since most of the conceived patient outcomes are well established from earlier studies, see below. The other conceived outcomes have not been described as outcomes before, and it may in fact be the case that they are more dependent on context.

With the phenomenographic research approach it was possible to bring out how the GPs experienced the outcomes close in time to the actual consultations. The study enlightened something they had not consciously reflected over and also brought to light less conventional thoughts about outcomes as well as their presumptions, when they were uncertain. In this way the study gave a comprehensive picture of possible consultation outcomes seen through the eyes of GPs.

It can be difficult to discern the outcome from a specific consultation. We asked about the latest consultation but cannot be sure of how it has been influenced by outcomes of a patient's preceding consultations.

In the groups the GPs supported each other. They valued rightly and wrongly and thereby themselves with regard to adopted professional norms, a perception of a collegial consensus. These aspects did not stand out in the individual interviews, where there was no group of colleagues to relate to. For this reason it was a strong point to have interviews both in groups and individually; the contrast became clearer. There was no other difference between the types of interviews regarding experiences of the other outcomes.

### The results

The GPs conceived outcomes in four ways; patient outcomes, GPs' self-evaluation, relationship building and change of surgery routines.

Regarding patient outcomes the conceived outcomes of the GPs were largely congruent with described and used indicators and measures. That attention was focused on patient satisfaction was obvious. It is not by chance so much evaluation is directed towards satisfaction. Or is it maybe the other way round? As satisfaction has been of importance in evaluations, the GPs will pay attention to it.

The GPs did not conceive the patient's health as a consultation outcome in the broad sense that health is measured in SF-36 [2] or EQ5D [3]. When they commented on patient cure or symptom relief due to the consultation they did not connect this to health.

They did not describe laboratory parameters as outcomes. When a control of these had been made it was the fact that a check-up had been performed that was considered to be the outcome. It is true that laboratory parameters have the paradoxical characteristic that though it is possible to measure a parameter with great exactness, you can never be sure of its significance for the individual patient.

PEI, Patient Enablement Instrument [5] is an instrument that in several ways is suited to measure GPs' consultation outcomes as it can capture both increased patient understanding and coping. But in our results the statements on support described mainly a support which was not expected to enhance either patient autonomy or coping. In some cases the GPs even experienced that support could be a hindrance for the patient in handling his real problems, which was exemplified in some sick-listing situations. To strike the balance between positive support, and support that is, in fact, holding the patient back, is not easy. Support that does not lead to a more autonomous or capable patient, but still does some good is neither taken up by PEI nor described in other ways.

Even though the GPs'conceptions of patient outcomes in most cases can be connected to indicators in established evaluation instruments, it is important to remember that these have limitations besides which the outcome becomes hidden among the questions about process and settings. The instruments are important as spot tests, for evaluation of interventions or to prove a certain outcome. But if they are to be regarded as the real outcome, without taking the outcome as a whole into consideration, there will be a risk that the efforts to evaluate and improve GPs' work in the future will be reduced to that which is easiest to measure with quality instruments [18]. In this study we found that the GPs themselves made an important complementary addition to the patient outcome evaluation, by making a self-evaluation, where they used internalized norms as a criterion for their assessment.

We were surprised by how strongly these internalized norms affected the GPs. The norms were perceived as a consensus between colleagues. The GPs have values and norms in common with other specialties, but they also have their own. It would be of interest to explore this further. Eliot Freidson has been into this subject when he studied how GPs evaluate mistakes in relation to "good clinical practice" – informal rules about what doctors should discover, understand and know [19]. If this consensus could be articulated and described, if GPs' self-evaluations, as in our groups, could be balanced by collegiality, the GPs' self-evaluation could be constructive and a source for professional development and quality assurance. To achieve such a development it would be necessary for GPs to take time to meet and discuss in groups, both spontaneously in everyday work and more systematically as in CME-groups [20].

Especially interesting, but also a complicated and delicate matter was self-evaluation when the will to follow the norm was in conflict with other perceived outcomes, as in the cases were the GPs directly contrary to their own conviction did something the patient had requested. Such conflicts can be harmful to both patients and GPs, and thus it is important have further light shed upon the subject.

Winefield has noted that GPs and patients are not satisfied with the same consultations [21]. Such differences can arise when the GP as opposed to the above situation in case of disagreement, does not submit to the patient, but sticks to what is perceived as professional consensus. Thus the patient and the GP do not assess the consultation after the same template. Accordingly the patients' evaluations cannot be regarded as being the only truth in consultation evaluations. This was discussed also by Fairhurst who found that doctors who had encountered somatising patients with psychosocial problems were more satisfied when they had helped the patient with what they perceived was the problem than when they had fulfilled the patient's own wishes[18].

The outcome status of this self-evaluation is reinforced by the fact that it will be at the back of the GPs' mind and influence his future consultations. Most likely the GPs will try to avoid situations where they feel that they are not good enough, neither when confronted with the patient, nor with their own professional norms.

The GPs attach importance to building a relationship with the patient, even if this could result in them acting in a way that they did not really approve of. The relationship is built gradually and is an important part of the Patient Centred Clinical Method [22]. The emphasis on being an expert not only on the disease but also on the patient is more important in general practice than in other specialties. The relationship is the base for being able to understand the patient as a person. Arborelius has found that patients highly appreciate their relation with their doctor [23]. Thus both GPs and patients perceive that the relationship is important, but the building of it has not been paid much attention to in evaluation.

The discovery of a need to change surgery routines was an obvious outcome. This reflects that the GP has not only the current consultation but also future consultations before his/her eyes when thinking of consultation outcomes. Besides, this category expresses a readiness to take on responsibility for her/his own organization. This responsibility can get lost in a big organization where the GP will be nothing but a little cog measured with a few indicators.

## Conclusion

The GPs conceived outcomes in four ways; patient outcomes, their self-evaluation, relationship building and change of surgery routines.

Taken separately, the patient outcomes conceived by the GPs are, to a great extent, congruent with those that had been brought up in outcome studies. Our study indicates that a comprehensive "GP-consultation outcome instrument", capturing the whole array of those outcomes, would be a great asset.

The GPs described the gradual work of relationship building as an outcome. Here they confirmed, from their own experience one of the cornerstones of the theories of general practice.

The GPs made self-evaluations in relation to internalized professional norms. It was striking how strongly the GPs wished to stick to professional norms. They assessed the feasibility of their surgery routines which showed that they felt responsibility also for their organization.

Patient outcomes can be evaluated with formalized procedures such as outcome or quality instruments, but these cannot be regarded as a complete evaluation of consultation outcomes. The assessment made by the practitioners themselves is intense, indispensible and will always be there. We believe that a deliberate interplay between these two forms of assessment may inspire the professional development of GPs.

## Competing interests

The authors declare that they have no competing interests.

## Authors' contributions

AA made the design of the study, carried out the interviews and transcribed them.

SOA and AA prepared the study.

AA and CR analysed the interviews. All three wrote the article.

## Pre-publication history

The pre-publication history for this paper can be accessed here:


